# Virtual Reality Cognitive Remediation for Mood disorders: RCT pilot study

**DOI:** 10.1192/j.eurpsy.2022.391

**Published:** 2022-09-01

**Authors:** L. Lipskaya-Velikovsky, D. Cohen, D. Livian-Carmel, G. Eger

**Affiliations:** 1 The Hebrew University of Jerusalem, Occupational Therapy, Jerusalem, Israel; 2 Beer Yaakov - Ness Ziona Mental Health Center, Occupational Therapy, Beer Yaakov, Israel; 3 Geha Mental Health Center, Occupational Therapy, Petah Tikva, Israel; 4 School of Health Professions, Sackler Faculty of Medicine, Tel Aviv University, Occupational Therapy, Tel Aviv, Israel; 5 Geha Mental Health Center, Psychiatry, Petah Tikva, Israel; 6 Tel Aviv University, Sackler Faculty Of Medicine, Tel Aviv, Israel

**Keywords:** Everyday functioning, Mood disorders, Cognitive remediation

## Abstract

**Introduction:**

Mood disorders interrupt well-being and participation in everyday activities through, among others, a mechanism of cognitive impairments. Ample evidence was found for cognitive remediation (CR) effectiveness in various mental health conditions. However, its contribution to improvement of functional outcomes in mood disorders was little investigated. Virtual Reality (VR)-based CR has a potential to overcome limitations by enabling training on daily-life tasks in ecological environments.

**Objectives:**

Test the effectiveness of VR-based vs standard CR for improvement of cognition, functional capacity and participation in daily-life activities in mood disorders.

**Methods:**

Twenty-two individuals (female: N=13, 59.1%; Age: M=39, SD=13.4) diagnosed with major depression or bipolar disorder were randomly assigned either to the standard or VR-based CR. The participants completed 6 half-an-hour sessions using the Functional Brain Trainer (Intendu©), a body-controlled, adaptive tool for training of inhibition, planning, working memory, shifting, self-initiation, persistence, and attention in functional tasks and environments. Standard assessments were used to evaluate cognition, functional capacity, mood symptoms and participation dimensions in pre-post design.

**Results:**

VR-based CR contributes to improvement in memory, executive functions and construction (2<Z<2.23, p<.05), functional capacity (Z=-2.44, p<.01) and satisfaction with participation (Z= -1.9, p<.01). Standard CR contributes to executive functions (Z=2.33, p<.05), and functional capacity (Z=-2.35, p<.05).

**Conclusions:**

This study provides initial evidence for contribution of CR to functional outcomes in mood disorders, with advantages of VR-based modality, suggesting the potential of CR to improve treatment outcomes and well-being in this population. Larger, controlled trials are needed to further expand evidence for VR-based CR effectiveness.

**Disclosure:**

No significant relationships.

